# A Privacy-Preserving Framework Using Homomorphic Encryption for Smart Metering Systems

**DOI:** 10.3390/s23104746

**Published:** 2023-05-14

**Authors:** Weiyan Xu, Jack Sun, Rachel Cardell-Oliver, Ajmal Mian, Jin B. Hong

**Affiliations:** Department of Computer Science and Software Engineering, The University of Western Australia, Perth, WA 6009, Australia; weiyan.xu@research.uwa.edu.au (W.X.); 23466264@student.uwa.edu.au (J.S.); rachel.cardell-oliver@uwa.edu.au (R.C.-O.); ajmal.mian@uwa.edu.au (A.M.)

**Keywords:** smart metering system, homomorphic encryption, trust boundary

## Abstract

Smart metering systems (SMSs) have been widely used by industrial users and residential customers for purposes such as real-time tracking, outage notification, quality monitoring, load forecasting, etc. However, the consumption data it generates can violate customers’ privacy through absence detection or behavior recognition. Homomorphic encryption (HE) has emerged as one of the most promising methods to protect data privacy based on its security guarantees and computability over encrypted data. However, SMSs have various application scenarios in practice. Consequently, we used the concept of trust boundaries to help design HE solutions for privacy protection under these different scenarios of SMSs. This paper proposes a privacy-preserving framework as a systematic privacy protection solution for SMSs by implementing HE with trust boundaries for various SMS scenarios. To show the feasibility of the proposed HE framework, we evaluated its performance on two computation metrics, summation and variance, which are often used for billing, usage predictions, and other related tasks. The security parameter set was chosen to provide a security level of 128 bits. In terms of performance, the aforementioned metrics could be computed in 58,235 ms for summation and 127,423 ms for variance, given a sample size of 100 households. These results indicate that the proposed HE framework can protect customer privacy under varying trust boundary scenarios in SMS. The computational overhead is acceptable from a cost–benefit perspective while ensuring data privacy.

## 1. Introduction

Smart metering systems (SMSs) are pivotal in modernizing the energy and resource sector, driving efficiency (e.g., energy distribution [1]), reliability (e.g., industrial applications [2]), and sustainability (e.g., water management [3]). Their novelty and contribution lie in providing accurate, real-time or near-real-time data on energy and resource consumption, enabling utilities to provide more reliable services, optimize system operations, identify inefficiencies, and support the integration of renewable energy sources (e.g., smart grid systems) [4,5]. Simultaneously, they contribute to empowering consumers to make informed decisions regarding energy use, leading to demand-side management and cost savings. By supporting infrastructure modernization, SMSs can reduce overall energy consumption, decrease carbon emissions, and advance the transition to a low-carbon and sustainable economy [6,7].

SMSs provide various features such as tracking real-time or near-real-time usage data [8], detection of abnormal usage [9], more accurate billing information [10], and the ability to share the collected fine-grained data with third-party analysts for more comprehensive analysis (e.g., dynamic price prediction [11]). For instance, smart water metering (SWM) systems can generate fine-grained temporal water usage data, which can be used to recognize user behaviors such as tapping, taking a shower [12], and gardening [13].

However, this raises serious privacy concerns, as malicious actors, including service providers, third-party analysts, or neighbors, can eavesdrop and analyze these data without user consent. Their motivations vary from identifying a specific customer to monitoring the billing information or analyzing user behaviors. Therefore, it is crucial to carefully evaluate the security implications of sharing customer data with related entities or outsourcing to third-party analysts in different scenarios. Some smart metering systems, such as SWM, may have sparsity in their fine-grained data sets, with a granularity of 10 s or even longer (i.e., the gap between water usages is usually large), which makes it easier to identify the user by recognizing their behavior and mapping to real-world activities. In addition, installing and implementing a smart metering system requires security considerations and standards [14]. Poor installation practices or unauthorized personnel installing the smart meters may leave them vulnerable to tampering or unauthorized access. Therefore, this study mainly concentrated on making a given SMS more privacy- and security-compliant but did not consider the side effects of the SMS (e.g., the broader implications of SMSs such as energy consumption or reduction, energy sources, sustainability, etc.).

Previous works have proposed various privacy-preserving technologies (e.g., differential privacy, federated learning, etc.) to address the issues above. For instance, Cardell-Oliver and Carter-Turner [15] proposed using k-anonymity and sampling with differential privacy guarantees to protect the privacy of the data sets for SWM. Taïk and Cherkaoui [16] used federated learning for household load forecasting in a smart grid. However, some limitations hinder their usage in practice. For example, differential privacy uses obfuscation techniques to guarantee security by adding noise, causing lower accuracy. Although federated learning transmits the updated gradients rather than the data, privacy leakage is still possible. Potential privacy-preserving techniques are discussed in Section 2. Moreover, these techniques need to be improved to satisfy generalized computational requirements and data features for different SMS scenarios. Therefore, there is a need for a privacy-preserving framework that considers these requirements.

This paper proposes a framework to protect data privacy for SMSs that considers trust boundaries (TBs) and computability using real-world data sets while providing privacy guarantees. The proposed framework uses homomorphic encryption (HE) as a primitive for security. HE can provide strong protection based on cryptography and data processing on encrypted data without first decrypting it. We have different scenarios with various trust boundaries (i.e., the entities involved may or may not trust each other) where entities can be assigned different public keys depending on their trust boundary. Currently, available HE implementations, such as Lattigo [17] and SEAL [18], support the CKKS scheme [19] (an HE scheme that supports fixed-point arithmetic operations). They provide primitive tools for building applications. Although Lattigo is written in Golang, it has a performance comparative to that of C/C++ libraries and compatibility with most operating systems and CPU architectures. Furthermore, it provides multiparty HE with a multiparty computation (MPC) protocol. Hence, our proposed framework uses Lattigo as the HE library for different SMS scenarios. In detail, we implemented simple computation metrics of summation and variance that third-party analysts can depend on when performing analytical tasks. Finally, we evaluated the proposed privacy-preserving framework for SMSs with real-world data and discussed some topics closely related to our framework.

Because we used HE as the primitive security method for SMSs, there are some challenges we need to consider. First, HE solutions need to be adaptive to different SMS scenarios under different trust boundaries and consider the attacks from adversaries at each point of the system. Second, the HE security parameter set has an important influence on the system security and performance. Therefore, we need to choose a proper security parameter set according to common HE security levels and the data size for HE, which is the slot number of the HE ciphertext. Third, key distribution is a crucial process for HE schemes, so we must design how to properly distribute the keys.

We summarize the contributions of the study below:

- We devekioed a privacy-preserving framework for smart metering systems utilizing homomorphic encryption.
- We utilized trust boundaries to analyze the roles of smart metering systems and help design the configurations for setting up homomorphic encryption, including the key distribution of homomorphic encryption.
- We evaluated the proposed framework with real-world data by measuring the performance, including the time consumption of HE operations, the time consumption of analysts’ metrics computation, and the disk consumption of HE keys.

## 2. Related Work

Smart metering systems (SMSs) are integral to modern resource management infrastructure, enabling the accurate measurement and monitoring of resource consumption in residential and commercial settings. However, SMSs have raised many security and privacy concerns [20], such as the fact that SMSs can be attacked through vulnerabilities to bring down the whole system and cause damage to customers and service providers. Particularly, SMSs can generate sensitive consumption data [8] that are used to provide valuable services such as load forecasting [16]. Meanwhile, many regulations and laws (e.g., GDPR [21]) exist as standards to protect data privacy. As a result, ensuring the security of smart metering systems has become a critical concern for SMSs.

### 2.1. Privacy Risk and Countermeasures of SMS

Cyber–physical systems (CPS), such as SMSs or IoT systems, combine physical and digital entities and generate enormous volumes of data. The data generated by SMSs can be used to infer customers’ presence [22] and for activity recognition, including short-term activities (e.g., tapping or taking a shower [12]) or long-term activities (e.g., gardening [13]). Priyadarshini [23] studied the optimal machine learning methods to reach a high accuracy of 98% in activity recognition using the data from smart wearables, which form an integral part of IoT systems.

Because behavioral patterns can be analyzed through activity recognition, privacy concerns are increasingly growing due to customers’ privacy exposure [24]. A simple solution is to use the energy stored in the households that can later reshape the usage profile. Li et al. proposed a Bayesian detection-operational privacy leakage metric [25] for evaluating privacy risk and studied an optimal privacy-preserving energy control strategy. Li et al. presented a way [26] of pruning vulnerable data and randomly selecting database proportions for publishing.

### 2.2. General Privacy-Preserving Techniques

Some general privacy-preserving techniques have been investigated to address the privacy issues of SMSs. One example is k-anonymity. Alsaid et al. applied the Mondrian algorithm to ensure k-anonymity by excluding personally identifiable information within a smart grid system [27], which achieves anonymization in nlog(n) time complexity. Stegelmann and Kesdogan proposed using pseudonyms implementing k-anonymization to avoid the service provider identifying a specific customer in a smart grid [28]. However, k-anonymity does not include randomization, and adversaries can still successfully make inferences if they already know some background knowledge. Therefore, SMSs are vulnerable to adversaries if they can monitor the customers for a long time.

Trusted execution environment (TEE), an isolated CPU space for secure computation, is a second choice. Karopoulos et al. chose TEE as trusted computing technology to protect cryptographic keys, sensitive data, and critical operations in the application of smart grids such as remote attestation [29]. Valadares et al. studied a trusted architecture solution based on TEE and other security mechanisms to protect data in IoT applications [30]. Although there is the advantage of low communication and computation costs, TEE has a cost for hardware and is highly reliant on hardware implementation. Moreover, TEE is suitable for the data federation rather than the data collection process.

MPC is a technique that utilizes an MPC protocol to make participants collaborate on computations over their inputs while keeping them private through protocols. An MPC protocol refers to the rules and procedures that enable each party to compute the function securely and privately. It defines how the parties interact with each other, how they share information, and how they combine their inputs to produce the desired result. MPC comes with the huge cost of communication overhead. Kirschbaum et al. presented a privacy-aware communication protocol [31] for smart grid systems based on secure multiparty computation, which allows the aggregation of consumption data of a group of smart meters without disclosing individual information. Although this solution can reduce the communication effort through a special initialization phase, this phase increases the system’s complexity, for example, increasing the overhead of pre-computation. Danezis et al. proposed an MPC scheme based on secret sharing [32] through which they examined the usage of complex functions on smart meters. However, the scheme requires more computing rounds and negatively affects the bandwidth and latency.

Differential privacy (DP) is also a widely used privacy-preserving method. However, by adding proper noise, there is always a balance between data utility and privacy protection. Assuming the water provider is honest and trustworthy, Cardell-Oliver and Carter-Turner proposed a solution by sampling differential privacy in SWM systems that use (ϵ,δ)-differential privacy for a sample of $N_{\beta }$ households [15], which significantly improved the differential privacy guarantees because smaller samples increase the adversary’s uncertainty about which households are in the sample. Gai et al. proposed a data aggregation scheme with local differential privacy (LDP) in smart grids [33] by discretizing and aggregating these data to meet the privacy guarantees of LDP and finally estimating the total or average power consumption after combining randomized responses.

HE is a promising technique that supports computations over encrypted data. Fully homomorphic encryption (FHE) [34] is regarded as the complete form of HE, as FHE supports an unlimited number of arbitrary computations for potentially complex applications. Tonyali et al. assessed the feasibility of FHE for smart grids by adapting one existing FHE scheme for advanced metering infrastructure (AMI). The data size and delay overheads were acceptable [35]. As FHE supports arbitrary function evaluation and an unlimited number of operations, it depends on bootstrapping to reduce the noise level of ciphertexts when the level of the computation circuit is deep. Considering privacy protection based on cryptography for the whole process, honest data collectors or third-party analysts are no longer needed; thus, HE is regarded as the most promising method.

## 3. System Model

A smart metering system (SMS) is advanced infrastructure utilizing modern communication and computing technologies to monitor, record, and manage resource consumption. In SMSs, multiple clients can be connected with multiple service providers responsible for data collection, analysis, load monitoring, and demand response. In addition, service providers may outsource the same tasks to multiple third-party data analysts.

**Figure 1:**
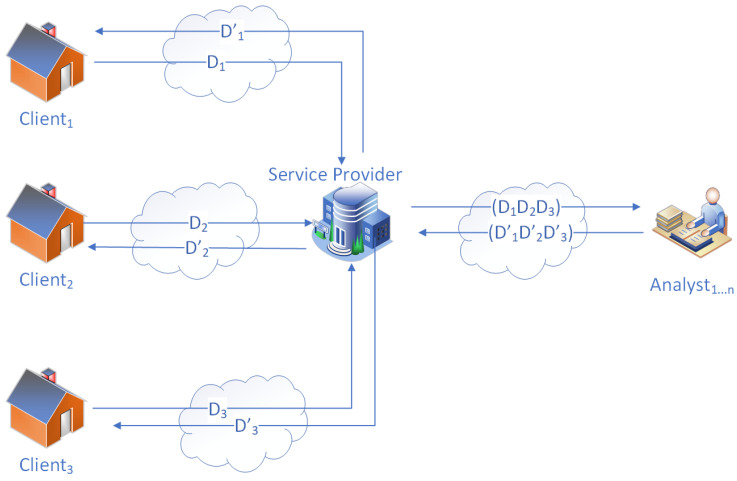
System model of SMS.

There can be varying models of SMS, but the simplest form of SMS is that there is only one service provider serving multiple clients. However, it may outsource its analytical tasks to multiple analysts, as depicted in Figure 1. For example, there is usually one water provider in an urban neighborhood containing dozens or hundreds of households where a smart water metering (SWM) system is used.

We make assumptions for the system model that only one service provider collects household consumption data and outsources the computations to third-party analysts.

### 3.1. Entities

There are mainly three types of entities involved in SMSs: (1) clients; (2) service providers; (3) third-party analysts. The clients are the ones that generate data. For example, smart water metering clients can generate water consumption data. Service providers collect data from clients and perform analytical metrics on the clients’ data but may outsource analyses to external analysts by sending clients’ data and collecting the outputs from the analysts to provide service for clients. Third-party analysts can compute different types of analytical results. For example, they can predict the dynamic prices for power utility services [11], calculate aggregations [36] for billing services, perform demand management [37], etc. Therefore, service providers may outsource such work to third-party analysts with the computational capabilities to perform these analyses.

### 3.2. Data Flow

There are two principal data flow pathways in a typical SMS configuration. The first pathway involves the data flow between clients and service providers. Generally, the process starts with the clients generating data, which the service providers collect to perform computations. The results of these computations are then used for billing the customers for the resources they consume and providing analytical metrics when requested. The data can be a series of raw data [12] on resource consumption during a specific period with an interval. The computations can be performed in-house or outsourced to third-party analysts.

The second data flow pathway involves a service provider and third-party analysts. Whenever the service provider cannot perform necessary computations due to a lack of expertise or resources, they can send the data to one or more third-party analysts to perform computations and generate the required results. Once the analysis is complete, the results are returned to the service provider.

### 3.3. Trust Boundary (TB)

Trust boundary (TB) [38] is a term used in computer science and security that describes a logical or physical boundary that divides domains with distinct levels of trust. It signifies the point at which an entity believes in another entity. TB can be a network perimeter, firewall, or other security measure distinguishing trusted and untrusted domains.

The TB illustrates the appropriate protection methods in the described SMS data flow scenarios. Essentially, it is a virtual scope in which entities are assumed to be honest, meaning they are not malicious and curious about the data. Thus, they would not violate data privacy or integrity by eavesdropping or tampering. Furthermore, no adversary can hack into these entities to corrupt data. As a result, the TB can clarify the design of the key management of distinct scenarios for SMSs with HE, including key generation and key distribution.

### 3.4. Computation Metrics

Because third-party analysts are assumed to have more computation capabilities than data owners, heavy computation tasks are outsourced to third-party analysts, and they can statistically analyze the data. For instance, they can calculate simple metrics, such as aggregation [36,39] (i.e., summation or average) to calculate the billing of the consumption. However, they can calculate more advanced metrics such as utilizing variance [40,41] for machine learning tasks to perform load prediction [42] and help resource providers perform demand management [37] to provide better service for customers. As how data are processed and transferred remains the same among entities, we applied summation and variance metrics as the computation metrics for simplicity. Other complex metrics, such as the ones mentioned above, can still be used in a practical environment.

We denote the set of households used in this paper as $H=\{h_{1},h_{2},\ldots \}$, where $h_{i}$ means the *i*th household in this set. We denote the consumption records of the *i*th household generated by its smart meter as $R\left(h_{i}\right)=\left\{r_{1}\left(h_{i}\right),r_{2}\left(h_{i}\right),\ldots \right\}$, where $r_{t}\left(h_{i}\right)$ is the *t*th record value of the *i*th household $h_{i}$. For example, for household 1, denoted as $h_{1}$, if we collect data with a granularity of 10 s (i.e., 8640 records/day), then the last one of a day’s recording would be $r_{8640}\left(h_{1}\right)$.

#### 3.4.1. Summation

One of the most widely used applications is the billing service or consumption monitoring, which requires the summation value as the basis of the computation formula. In detail, the summation of the daily, monthly, or quarterly energy consumption collected by smart meters can reveal customers’ behaviors and habits. Moreover, the computation of the summation function is straightforward. Hence, using HE to handle this problem is a good choice. The summation equation to calculate the sum value of the consumption records of the *i*th household per day is shown in Equation (1). Here, $d_{1}$ and $d_{n}$ define the period in $R(h_{i})$ iin which the summation equation is applied (e.g., the measurements for the billing period).

(1) $$\sum_{t=d_{1}}^{d_{n}}r_{t}\left(h_{i}\right)$$

#### 3.4.2. Variance

Variance is a statistical metric that reveals the data differences. It can be applied to one household and households in a group (e.g., a neighborhood). It relies on the sum of the actual samples and the sum of the squares. The equation to calculate the variance value of the consumption records of the *i*th household in the period $\left[d_{1},d_{n}\right]$ requires the calculation of the average consumption, which is shown in Equation (2), where $R^{\prime }\left(h_{i}\right)\subseteq R\left(h_{i}\right)$ denotes the recordings between $\left[d_{1},d_{n}\right]$. Then, the variance calculation is shown in (3).

(2) $$E\left(R^{\prime }\left(h_{i}\right)\right)=\sum_{t=d_{1}}^{d_{n}}\frac{r_{t}\left(h_{i}\right)}{n}$$

(3) $$V\left(R^{\prime }\left(h_{i}\right)\right)=\sum_{t=d_{1}}^{d_{n}}\frac{{\left(r_{t}\left(h_{i}\right)-E\left(R^{\prime }\left(h_{i}\right)\right)\right)}^{2}}{n}$$

## 4. Threat Model

**Figure 2:**
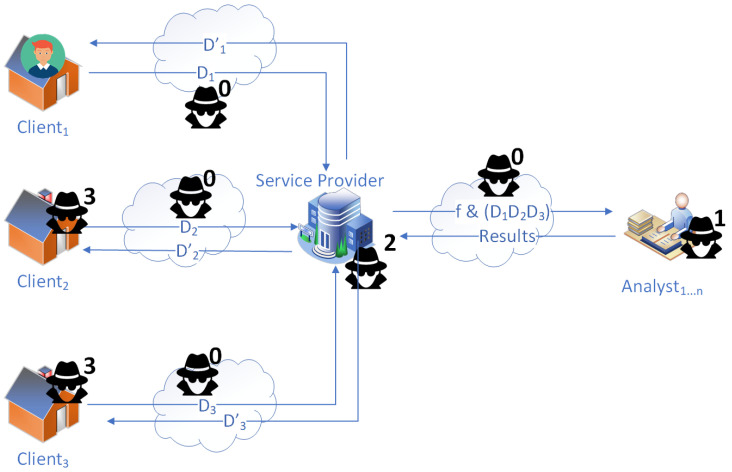
Threat model of SMS.

There are four kinds of adversaries in the data flow model of SMSs above, as shown in Figure 2. We label them from type 0 to 3 (the icon of a person wearing a black hat, high collar windbreaker, and sunglasses) and explain their threats.

Adversaries of type 0 cannot only steal data from the communication channels intercepted for malicious usage but can also corrupt data. Moreover, this kind of adversary can use multiple attack methods at any point in the communication channel as external attackers, causing enormous threats to the system. For instance, the most famous is the man-in-the-middle (MitM) attack [43], which can intercept and modify data between communications of two endpoints. Other attacks [44] include session hijacking, eavesdropping, denial of service (DoS) attacks, etc.

The other three types can also steal data or, in most cases, eavesdrop on data. However, they do not tamper with the data like type 0 adversaries because of their business roles in the system and that tampering could be easily detected if they corrupt the data. Hence, we can summarize them as honest but curious threats in SMSs.

Adversaries of type 1 are at the points of third-party analysts who receive consumption data from the service provider, perform analytical tasks, and return results to the service provider. Because they are business organizations with reputations, we can assume that they are at most honest and curious but not malicious. Concerning this assumption, they are not expected to tamper with the data but can eavesdrop on or steal it. Furthermore, if data are not protected using privacy-preserving techniques, adversaries of type 1 can use the data for privacy-violating purposes, such as activity recognition of user behaviors (e.g., taking a shower [12] and gardening [13]).

Adversaries of type 2 are at the points of the service provider, who collects consumption data from each household, pushes the data to analysts’ sides, retrieves analysis results, and transmits the appropriate results to each household. The service provider can also be regarded with a business reputation as a third-party analyst. Therefore, it is also assumed to be at most honest but curious, meaning that they have a chance to spy on or steal data but can not corrupt it.

Adversaries of type 3 are at the points of households that generate consumption data and receive the analytical reports from the service provider. In contrast to the analysts and the service provider, adversaries of type 3 are completely untrustworthy such that they can tamper with the data, not just eavesdrop on or steal it. For example, an adversary of type 3 can launch data injection attacks to manipulate locational marginal prices to obtain economic benefits [45]. Hence, being honest but curious will not apply to adversaries of type 3 as they may try to modify the consumption data of their neighbors nearby.

## 5. SMS Framework with Homomorphic Encryption

### 5.1. HE as Security Basis

Although there is a certain amount of computational and communication cost for SMSs when HE is adopted, it is still a promising candidate for privacy protection. The biggest advantage of HE is that it can not only protect data based on cryptography but also offer the capability of computations over ciphertext with arbitrary computational metrics when fully homomorphic encryption (FHE) is used. Thus, as a basic security primitive, it can cover different scenarios and vulnerable points of privacy leakage in the whole data flow of SMSs.

Furthermore, HE is a generalized technique based on encryption. For example, in SWM systems, user data can be sparse under fine-grained granularity, and HE can fit well into it. Hence, we can use HE for any SMS. After applying HE, adversaries cannot infer any useful information from the ciphertexts or from the public and evaluation keys generated by HE schemes based on its cryptographic mathematics, as detailed in Section 6.1. Additionally, the same plaintexts are encrypted into different HE ciphertexts with the help of randomized noise. HE has shown great generalization ability for data protection and a strong security guarantee. Furthermore, it avoids the possibility of side-channel attacks based on statistical analysis, complex machine-learning techniques, and other attacks, such as a chosen plaintext attack.

In HE, the secret key must be kept safe without exposure.

### 5.2. Certificate Authorities

A certificate authority (CA) is a trusted third-party entity in a public key infrastructure (PKI) used to verify the identity of individuals, companies, or devices on the Internet. They are normally reputable external organizations with prestige. A certificate issued and signed by a trusted CA to an entity contains crucial information about the certificate holder’s identity and its public key. Because HE depends on the keys it generates to protect data privacy, the key distribution of HE schemes is important to keep the whole system secure. Hence, we combine CA to issue certificates as an important step for key distribution in HE schemes to avoid adversaries of type 0, which is discussed in Section 6.4.

### 5.3. SMS Scenarios with Trust Boundary (TB)

Concerning the risks described in Section 4, we describe three necessary scenarios to be handled here. Scenario 1 is typical because the service provider normally deploys smart meters. Therefore, we can trust the service provider. Scenario 2 can also be reasonable, as privacy risks such as activity recognition exist in some service providers, and regulations such as GDPR [21] severely constrain data processing. Thus, service providers are greatly motivated to adopt varying methods to protect data privacy. This is where Scenario 2 comes in and can be achieved using HE. In Scenario 3 there is a possibility that a neighbor in a community can be malicious and will try to tamper with the data of other households.

#### 5.3.1. Scenario 1: Households and Service Provider in the Trust Boundary

The first scenario aims to eliminate adversaries from the analysts’ viewpoint (i.e., adversaries of type 1), who can eavesdrop. As initialization, entities must set up a secure communication channel (using a protocol such as TLS) between them. To achieve this, the service provider and each analyst must generate their public/private key pairs and request a certificate from the CA.

**Figure 3:**
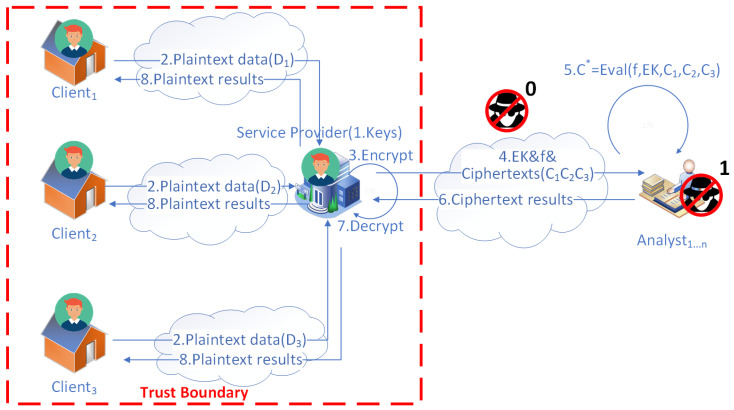
The proposed framework of SMS for Scenario 1.

After the framework has set up secure communication channels, the following steps describe the framework process depicted in Figure 3.

1. The service provider generates a set of keys (secret, public, and evaluation keys) for HE and keeps the secret key safe.
2. Each household sends its consumption data to the service provider through the secure communication channel.
3. After receiving each household’s data, the service provider encrypts each household’s consumption data to ciphertexts with the HE public key.
4. The service provider determines the computation metrics and sends them together with the collected ciphertexts and the evaluation keys to a list of third-party analysts of cooperation.
5. After receiving the ciphertexts, evaluation keys, and computation metrics, the analysts utilize them to perform homomorphic computations over the ciphertexts to obtain the ciphertext results $C^{*}$.
6. The analysts return the ciphertext results to the service provider.
7. The service provider decrypts the ciphertext results to plaintext results using its secret key.
8. The service provider sends the plaintext results to each household through the secure communication channel (TLS), which is encrypted by the TLS session key while in transit.

In Figure 3, the communication channels between each household, the service provider, and each third-party analyst are enhanced by introducing CA and certificates. After verifying its certificate, the identity of the service provider and analysts can be trusted. With a trust boundary containing all the households and the service provider, data privacy is guaranteed by HE. Furthermore, data are encrypted by HE when transferred from the service provider to the third-party analysts and when homomorphic computations occur on the analysts’ sides. As a result, adversaries of type 1 who want to eavesdrop can be eliminated. They can not obtain valuable information, such as inferring with the identity of any household or identifying any activity from the corresponding ciphertexts.

**Figure 4:**
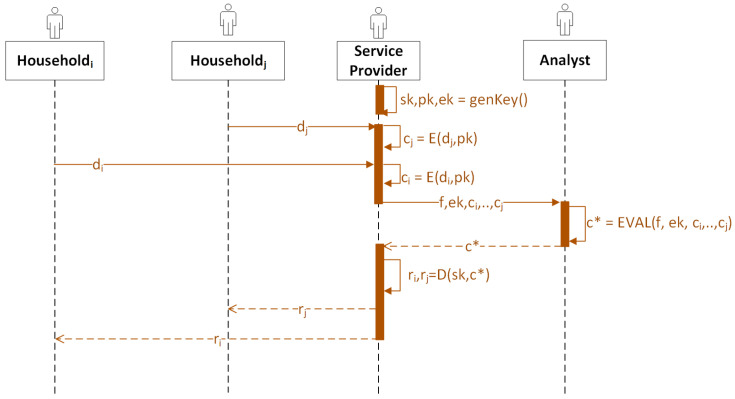
The sequence diagram for Scenario 1. *E* stands for encryption, *D* stands for encryption, EVAL stands for evaluation, $d_{i}$ stands for the consumption data from $household_{i}$, $c_{i}$ stands for the ciphertext encrypted from $d_{i}$, $c^{*}$ stands for the ciphertext result, $r_{i}$ stands for the plaintext result to $household_{i}$, and *f* stands for the homomorphic functions chosen by the service provider that third-party analysts perform.

To better illustrate the data flow of the entities in Scenario 1, we provide a sequence diagram in Figure 4.

This scenario has the highest requirements for system assumption because it requires all households and the service provider to be honest and not curious inside TB. This scenario is possible. Because normally, it is the service provider who deploys the smart meters for each household, and each house can trust the service provider to be honest and not curious.

#### 5.3.2. Scenario 2: Households in the Trust Boundary

The second scenario aims to eliminate adversaries from the analysts’ point (i.e., adversaries of type 1) and adversaries from the service provider’s point (i.e., adversaries of type 2), who can eavesdrop. As initialization, entities must set up a secure communication channel (TLS) between them. To achieve this, the randomly selected main household, the service provider, and each analyst must generate their public/private key pairs and request a certificate from the CA.

**Figure 5:**
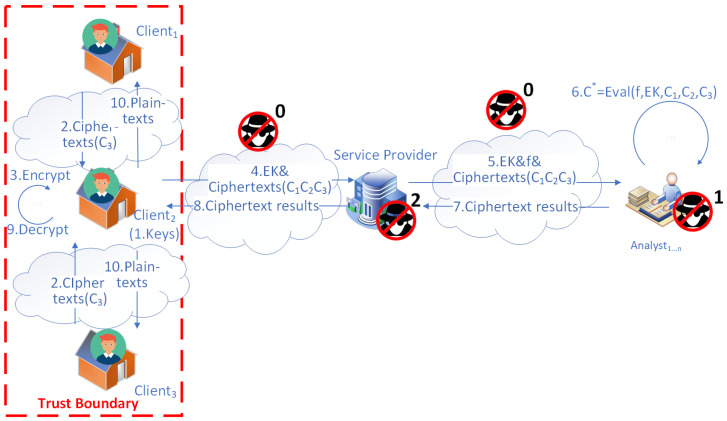
The proposed framework of an SMS for Scenario 2.

After the framework has set up secure communication channels, the following steps describe the framework process depicted in Figure 5.

1. The randomly selected main household generates a set of keys (secret, public, and evaluation keys) for HE and keeps its secret key safe.
2. Other households send plaintext data to the main household through the secure communication channel.
3. After receiving the plaintext data, the main household encrypts them into ciphertexts with the public key.
4. The main household sends the ciphertexts it collects to the service provider, along with the evaluation keys it generated in step 1.
5. The service provider chooses the computation metrics and sends them together with the ciphertexts and the evaluation keys to a list of cooperative third-party analysts.
6. After receiving the ciphertexts, evaluation keys, and computation metrics, the analysts utilize them to perform homomorphic computations over the ciphertexts to obtain the ciphertext results $C^{*}$.
7. The analysts return the ciphertext results to the service provider.
8. The service provider returns the ciphertext results to the main household.
9. The main household decrypts the ciphertext results to plaintext results using its secret key.
10. The main household transfers the plaintext results to other households through the secure communication channel (TLS), which is encrypted by the TLS session key in transit.

Similarly, in Figure 5, the communication channels between each household, the service provider, and each third-party analyst are enhanced by introducing a CA and certificates. After verifying its certificate, the identities of the main household, the service provider, and analysts can be trusted. With a trust boundary containing all the households, data privacy is guaranteed by HE. Data are encrypted by HE when transferred from each household to the main household and to the analysts through the service provider. Then, homomorphic computations occur on the analysts’ sides. As a result, adversaries of type 1 at the analysts’ points and adversaries of type 2 at the service provider’s point who want to eavesdrop can be eliminated. They can not obtain any useful information, such as the identity or activities of any household from the corresponding ciphertexts.

**Figure 6:**
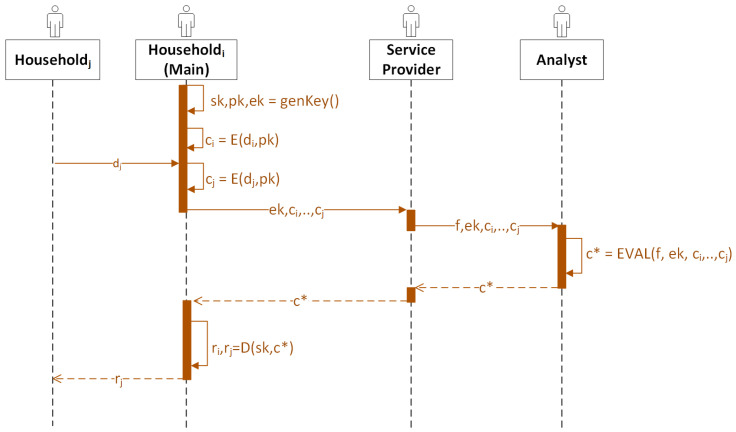
The sequence diagram for Scenario 2. *E* stands for encryption, *D* stands for decryption, EVAL stands for evaluation, $d_{i}$ stands for the consumption data from $household_{i}$, $c_{i}$ stands for the ciphertext encrypted from $d_{i}$, $c^{*}$ stands for the ciphertext result, $r_{i}$ stands for the plaintext result to $household_{i}$, and *f* stands for the homomorphic functions chosen by the service provider that third-party analysts perform.

To better illustrate the data flow of the entities in Scenario 2, we provide a sequence diagram in Figure 6.

This scenario has a trust boundary containing each household but excluding the service provider. This is also possible: although the service provider deploys smart meters, a curious employee who tries to eavesdrop on customers’ data might be working for the service provider. This is what regulations such as GDPR [21] are also trying to resolve.

#### 5.3.3. Scenario 3: Only One Household in the Trust Boundary

The third scenario aims to eliminate adversaries at the points of the analyst, service provider, and other households (i.e., adversaries of type 1, type 2, and type 3). As initialization, entities must set up a secure communication channel (TLS) between them. To achieve this, the service provider and each analyst must generate their public/private key pairs and request a certificate from the CA.

**Figure 7:**
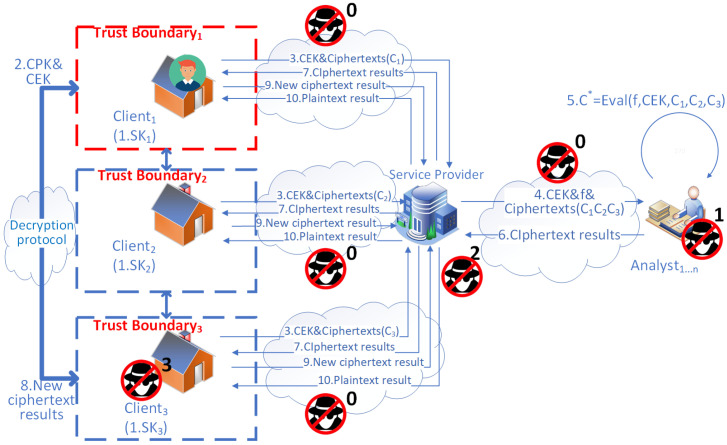
The proposed framework of an SMS for Scenario 3.

After the framework has set up secure communication channels, the following steps describe the framework process depicted in Figure 7.

1. Each household generates its own secret key ($SK_{i}$) and keeps it safe.
2. With the help of an MPC protocol, the collective public key (CPK) is generated by combining the local share of the computation result of each household. The collective evaluation keys (CEKs) are also generated. In this way, each household jointly determines the CPK and CEK, not needing to expose its secret key.
3. Each household encrypts the consumption data to ciphertexts with the CPK and sends them with the evaluation keys to the service provider.
4. The service provider receives each household’s ciphertexts and evaluation keys. Then, it chooses the computation metrics and sends all these data to the analysts, including the computation metrics, the evaluation keys, and ciphertexts from households.
5. After receiving the ciphertexts, evaluation keys, and computation metrics, the analysts utilize them to perform homomorphic computations over the ciphertexts to obtain the ciphertext results $C^{*}$.
6. The analysts return the ciphertext results to the service provider.
7. The service provider returns the ciphertext results to each household.
8. Each household applies an MPC protocol to turn the ciphertext results into new ciphertext results encrypted by the service provider’s public key (generated for secure communication) by combining the local share of the computation result of each household.
9. The new ciphertext results are pushed to the service provider, who can decrypt them to plaintext results using its secret key (generated for secure communication).
10. The service provider transfers the plaintext results to each household through the secure communication channel (TLS), which is encrypted by the TLS session key in transit.

In Figure 7, the communication channels between each household, the service provider, and each third-party analyst are enhanced by introducing a CA and certificates. After verifying its certificate, the identity of the service provider and analysts can be trusted. With a trust boundary containing only one entity (i.e., the household) for the specific household, data privacy is guaranteed by HE. Data are encrypted by HE when transferred from this household to the third-party analysts through the service provider and when homomorphic computations occur on the analysts’ sides. As a result, adversaries of type 1, type 2, and type 3 who want to eavesdrop can be eliminated. They cannot obtain valuable information, such as the identity or activities of the specific household from the corresponding ciphertexts.

Even when collectively computing the CPK and CEK, privacy is protected because we leverage the multiparty homomorphic encryption (MHE) proposed by Mouchet et al. [46], which has a multiparty computation (MPC) protocol to protect the data privacy of each household on its own. Moreover, they reduced the communication complexity from quadratic to linear concerning the number of parties. This way, each household efficiently utilizes its secret key and computes its share of the collective keys for usage (e.g., encryption and evaluation).

**Figure 8:**
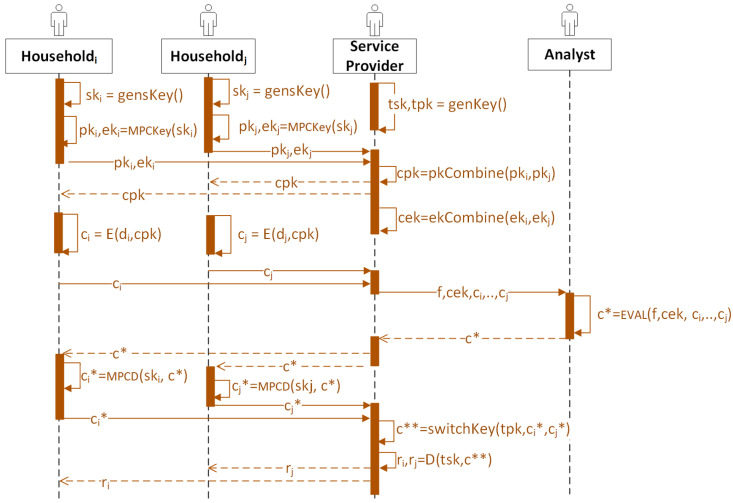
The sequence diagram for Scenario 3. cpk and cek stand for the collective public key and collective evaluation keys generated through MPC protocols; $sk_{i}$ stands for the secret key generated and used by $household_{i}$, tpk and tsk stand for the public and secret key pair generated and used by the service provider, respectively; *E* stands for encryption; *D* stands for decryption; EVAL stands for evaluation; $c_{i}$ stands for the ciphertext encrypted from the consumption data; $c^{*}$ stands for the ciphertexts result in the array after homomorphic computations; $c_{i}^{*}$ stands for the computation share of $household_{i}$; $c^{**}$ stands for the ciphertexts result in the array after switching public key from cpk to tpk; $r_{i}$ stands for the plaintext result to $household_{i}$; and *f* stands for the homomorphic functions chosen by the service provider that third-party analysts perform.

To better illustrate the data flow of the entities in Scenario 3, we provide a sequence diagram in Figure 8.

This scenario has a trust boundary containing only the household itself. This is the most common in practice as we need to rely on the honesty and the lack of curiosity on the part of the service provider, the analysts, and even other households. Because after eliminating the privacy risks of the service provider and third-party analysts, the neighbors near the household may try to eavesdrop on the data. Hence, each household keeps its secret key to encrypt data and protect privacy. Therefore, this method can provide a practical solution for user data privacy preservation.

### 5.4. HE Encryption and Decryption Algorithms of the Framework

#### 5.4.1. HE Key Generation for Algorithms

As there is only one pair of public and secret keys in Scenarios 1 and 2; they can be categorized into single-party homomorphic encryption. The key generator in Scenario 1 is the service provider, while in Scenario 2, the randomly selected main household serves as the key generator. The key generator produces the target public key, the target secret key, the relinearization key, and the rotation key.

In Scenario 3, each household has a secret key to keep their privacy. The other HE keys are collectively generated among the households via an MPC protocol. The collective public key, the collective relinearization key, and the collective rotation key are generated through the MPC protocol. The service provider behaves as the node for information combination, while each household computes its share of information needed for the keys’ generation. Finally, the keys are generated by the service provider.

#### 5.4.2. HE Encryption Algorithm

The consumption data are then encrypted into ciphertexts by the service provider in Scenario 1. In Scenario 2, the randomly selected main household encrypts the consumption data into ciphertexts. They both use the target public key tpk as the encryption key pk in the Algorithm 1.
**Algorithm 1** HE encryption algorithm.1:Input *N*: the number of the households2:Input *P*: the plaintext consumption data array of the households3:Input encryptor: the object that performs encryption4:**for** index=1,2,…,N **do**5:      data←P[index]6:      c←encrypt(data,encryptor,pk)7:      push *c* into ciphertexts array8:**end for**9:Output ciphertexts array

The encryption process in Scenario 3 is multiparty homomorphic encryption, which is the same as single-party homomorphic encryption, except that each household uses the collective public key cpk as the encryption key pk to encrypt the consumption data.

#### 5.4.3. HE Decryption Algorithm

The ciphertext results are then decrypted into ciphertexts at the service provider side in Scenario 1. In Scenario 2, the randomly selected main household is responsible for decrypting the ciphertext results into plaintext results. The decryptor uses the target secret key sk as the decryption key in Algorithm 2.
**Algorithm 2** HE decryption algorithm for Scenarios 1 and 2.1:Input sk: the secret key for the decryption2:Input ciphertexts: the ciphertexts array3:Input *N*: the number of the households4:Input decryptor: the object that performs decryption5:**for** index=1,2,…,N **do**6:      $c^{*}\leftarrow ciphertexts\left[index\right]$7:      $data\leftarrow decrypt(c^{*},decryptor,sk)$8:      push data into plaintexts array9:**end for**10:Output plaintexts array

For Scenario 3, the scenario of multi-party homomorphic encryption, the service provider can not directly decrypt the ciphertexts encrypted with the collective public key cpk. Thus, the decryption process first requires the SwitchKeyViaMPC function over an MPC protocol to convert the ciphertexts to the form encrypted with the target public key pk so that the service provider can directly decrypt these ciphertexts using the target secret key sk, which are shown in Algorithm 3.
**Algorithm 3** HE Decryption Algorithm for Scenario 31:Input pk: the target public key for the new ciphertexts after converted from an MPC protocol2:Input sk: the target secret key for the decryptor3:Input *N*: the number of the households4:Input *H*: the households array, each household keeping its secret key5:Input decryptor: the object that performs the decryption6:**for** index=1,2,…,N **do**7:      $c^{*}\leftarrow ciphertexts\left[index\right]$8:      $c^{**}\leftarrow SwitchKeyViaMPC\left(params,pk,c^{*},H\right)$9:      $data\leftarrow decrypt(c^{**},decryptor,sk)$10:     push data into plaintexts array11:**end for**12:Output plaintexts array

### 5.5. Computation Metrics’ Implementation

We describe below how third-party analysts perform homomorphic encryption using the summation and variance metrics in Section 3.4. For simplicity, we provide depictions using an HE ciphertext with four slots. However, in practice, there are many more slots in a ciphertext, which depends on *N*, one of the security parameters of HE schemes, and is often assigned as a power of two.

We describe the functions applied in the algorithms below. The function sumEachElement is used to sum each element of the ciphertext *c* so that each element of the new ciphertext *c* has a value that is equal to the sum of all elements of the original ciphertext. The function averageBy reduces each element of the ciphertext *c* by *n* times. The function Add involves adding two ciphertexts together and returning the sum ciphertext. The function Multiply acts to multiply two ciphertexts together and return the product ciphertext.

#### 5.5.1. Summation

We illustrate the summation computation in Algorithm 4.
**Algorithm 4** Summation algorithm for ciphertexts.1:Input ciphertexts: the ciphertexts array transferred from the service provider2:Input evaluator: the object that performs homomorphic computations3:**for** index=1,2,…,N **do**4:      c←ciphertexts[index]5:      evaluator.sumEachElement(c)6:**end for**7:Output ciphertexts back to the service provider

**Figure 9:**
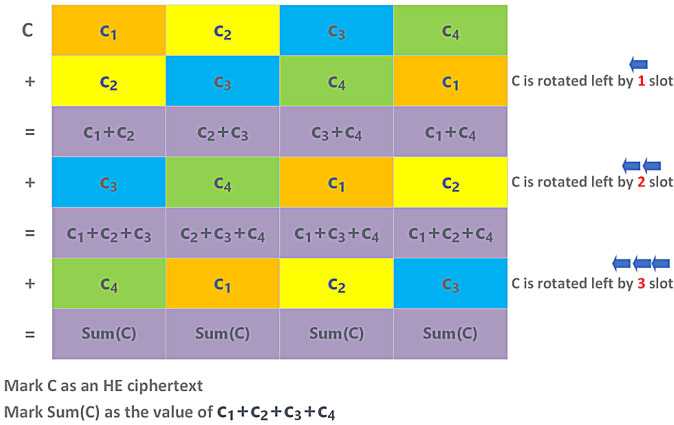
The process of the HE summation metric over ciphertext.

In detail, the process of the sumEachElement function is shown in Figure 9. Each element $c_{i}$ of the ciphertext *C* contains the summation value of the original ciphertext after the homomorphic computations.

#### 5.5.2. Variance

We illustrate the variance computation in Algorithm 5.
**Algorithm 5** Variance algorithm for ciphertexts.1:Input *N*: the number of the household2:Input rowCount: the count of records per day of each household3:Input ciphertexts: the ciphertexts array transferred from the service provider4:Input evaluator: the object that performs homomorphic computations5:**for** index=1,2,…,N **do**6:      c←ciphertexts[index]7:      $c_{copy}\leftarrow copy\left(c\right)$8:      evaluator.sumEachElement(c)9:      c.averageBy(rowCount)10:    c←evaluator.Multiply(c,−1)11:    $c\leftarrow evaluator.Add(c,c_{copy})$12:    $c^{\prime }\leftarrow evaluator.Multiply\left(c,c\right)$13:    $evaluator.sumEachElement(c^{\prime })$14:    $c^{\prime }.averageBy\left(rowCount\right)$15:**end for**16:Output ciphertexts array back to the service provider

The variance computation involves addition and multiplication operations. Based on the summation of a ciphertext, it is easy to compute the variance of the original ciphertext.

**Figure 10:**
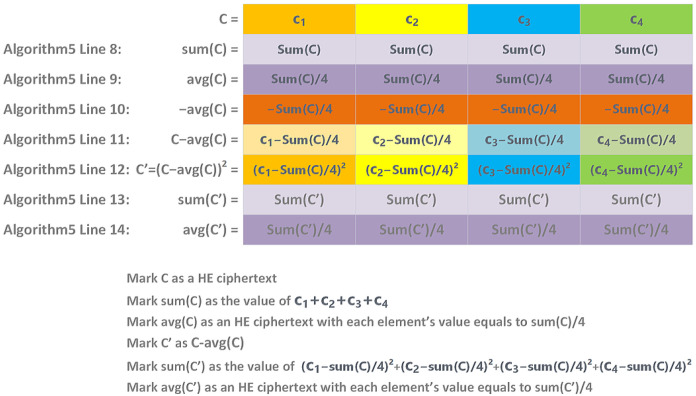
The process of the HE variance metric over ciphertext.

In detail, we depict each key step in Algorithm 5 mapped into Figure 10. After the homomorphic computations, each slot of the ciphertext contains the variance value of the original ciphertext.

## 6. Security Analysis

As we utilize the HE scheme (CKKS [19]) as the basis of the proposed privacy-preserving framework, our proposed framework significantly relies on the security of the HE cryptographic foundation, the choice of the security parameter set, and the key distribution of the HE scheme. So, we present different perspectives of security considerations.

### 6.1. Cryptography Security of HE

The security of an HE scheme (i.e., CKKS [19] in this paper) is based on the hardness of the ring learning with errors (RLWE) problem [47], a variant of the learning with errors (LWE) problem, which is regarded as a computationally difficult problem where adversaries are trying to recover the secret coefficients, generating noisy samples when a set of samples of noisy linear equations on a polynomial ring over finite fields is provided. It is difficult to solve the RWLE problem with even a quantum computer [48]. Thus, the RLWE problem can be used to build secure schemes with quantum resistance and behave as the cryptographic foundation for homomorphic encryption, just as the large number factorization problem has provided for the RSA, a public key cryptographic algorithm in use since 1977.

### 6.2. Cryptanalysis of HE Scheme CKKS

A security model based on indistinguishability under the chosen plaintext attack (IND-CPA) exists. CKKS, among many HE schemes, can be proven to satisfy IND-CPA security [49] based on the learning with errors (LWE) hardness assumption. Li and Micciancio [50] proposed a stronger security notion called IND-CPA+, which extends the notion of IND-CPA and to combat passive attacks. They pointed out that the weakness of the CKKS scheme against IND-CPA+ adversaries mainly comes from the possibility of recovering the secret key based on the linearity of the decryption function. Additionally, the HE library we use (Lattigo [17]) was updated to mitigate IND-CPA+ attacks by applying a rounding strategy to attach a proper error to make CKKS more secure.

### 6.3. Security Parameter Set of HE Scheme CKKS

Although HE has solid secure guarantees derived from cryptography, in practice, for an HE implementation (e.g., CKKS), the parameter set that ensures the scheme’s security must also be carefully chosen. Specifically, there are three parameters [51] related to the security of HE schemes based on RLWE: (1) *n* (the dimension of a specified ring *R*), which has an impact on both security and scheme performance, with the scheme security increased at a larger *n*, but the performance decreased at a larger *n*; (2) a ciphertext modulus *q* that also influences both security and scheme performance by decreasing them at a larger *q* for a fixed *n*; (3) the standard deviation of the error distribution σ, which results in better security at a larger σ. Consequently, there is a need for researchers, companies, and government agencies to perform experimentations to realize a standardized choice of security parameter sets. Chase et al. [52] summarized security parameter sets concerning different security levels (i.e., 128-bit, 192-bit, and 256-bit). Based on that paper, we chose the security parameter set described in Section 6.3 for the framework to achieve a 128-bit security level.

### 6.4. Security of HE Key Distribution

As mentioned, the secret key for HE must be kept safe without exposure. In contrast, the public and evaluation keys must be shared with anyone who wishes to encrypt plaintexts or perform homomorphic computations over ciphertexts. However, a risk of privacy leakage is still caused by key exchange. For instance, in the first scenario mentioned in Section 5.3.1, a faked public key can be pushed to a household by an adversary who intercepts the communication between the household and the service provider. Then, the consumption data of the household would be encrypted by that faked public key and easily decrypted by the adversary. Likewise, homomorphic operations can be influenced by the faked evaluation keys when third-party analysts receive them from the faked service provider. So, it is necessary to ensure the validity of keys by key exchange.

In detail, key exchanging refers to sharing a key securely between two parties through a protocol without the secret information being intercepted or tampered with by an adversary. For example, Diffie-Hellman key exchange [53], or its variant ECDH [54], is practically served for this purpose. Similarly, key distribution is a broader concept meaning distributing keys to multiple parties to communicate securely and efficiently where a trusted party for key management is commonly involved. Typical key distribution systems include Kerberos [55] and a highly secure and scalable public key infrastructure (PKI) [56].

We adopted PKI to ensure key distribution of the privacy-preserving framework by introducing a certificate authority (CA) to prove the identity of each party so that each entity in the framework can verify the validity of the public and evaluation keys from the key generator if needed.

Adversaries of type 0 cannot threaten the privacy or integrity of the data during transmission, as a secure cryptographic protocol such as transport layer security (TLS) is used to encrypt and verify the integrity of any data exchanged between two parties [57].

We focused on protecting privacy and security at three points against adversaries: third-party analysts against adversaries of type 1, the service provider against adversaries of type 2, and the clients’ points against adversaries of type 3.

The security analysis of HE key distribution for the three scenarios is provided below.

- In Scenario 1, the service provider is inside the trust boundary. Hence, it is honest and not curious. It is safe for all households to trust the service provider and transfer the plaintext data to the service provider through secure communication (i.e., TLS protocol). In this way, there is no need for the HE public key to be distributed. So, the privacy of each household can never be violated. The evaluation keys need to be transmitted from the service provider to third-party analysts with ciphertexts through secure communication.
- In Scenario 2, it is unsafe to trust the service provider because it is outside the trust boundary. Hence, households cannot trust the keys generated by the service provider for its possibility of curiosity. Because all households are inside the trust boundary, a randomly selected household can be the key generator. It is safe for other households to trust the main household and transfer the plaintext data through secure communication (i.e., TLS protocol). In this way, there is no need for the HE public key to be distributed. So, the privacy of each household can never be violated. Additionally, the evaluation keys need to be transmitted from the main household to third-party analysts through the service provider with the ciphertexts through secure communication.
- In Scenario 3, each household is protected in its trust boundary, not sharing the same trust boundary with others and not trusting the service provider, third-party analysts, or other households. In this way, privacy and security can be maximized. We applied multiparty homomorphic encryption [46] with an underlying multiparty computation (MPC) protocol to compute each household’s key share and securely join them as the combined collective key, for instance, the collective public key for each household to complete data encryption. Thus, they do not need a certificate verification process.

## 7. Evaluation

### 7.1. Data Collection

We used a real-world SWM data set from project DAIAD [58] containing the time series of smart water meter consumption. It contains the SWM time series for 1007 consumers of water utility AMAEM. The data set includes 16,857,056 measurements, which amounts to 16,739 per user. Because we focused on the framework overheads, we could use this data set to group 8640 water consumption records for each user as the evaluation basis.

### 7.2. Environment Setup

We have set up a GitHub repository for the project’s code here at https://github.com/cyberhermitcrab/lattigo (accessed on 29 March 2023). The code was tested via two devices. We used a Windows laptop with a 1.90 GHz AMD Ryzen 7 5800U-rocessor and 16 GB RAM for the service provider’s and third-party analysts’ computation tasks. We used a MacBook Pro with a 2.3 Ghz Intel Core i5 Processor and 4 GB 1333 MHz DDR3 RAM for households’ computation tasks.

We applied the HE scheme CKKS [19] for fixed-point arithmetic. Moreover, we chose Lattigo [17] as the code library, written in GO language, with excellent performance and concurrent operations. Moreover, it supports multiparty homomorphic encryption as an extension, with a multiparty protocol for computation for each household.

We tested with 8640 records for each household. According to the Homomorphic Encryption Standards group, we chose the security parameter set for the security level of 128 bits. Hence, we used the below set of security parameters to ensure the hardness of the scheme with the security level of 128 bits: $Log\left(N\right)=14,Log\left(Q\right)=438,Log\left(Slots\right)=\;14,DefaultScale=2^{34}$.

### 7.3. Results

#### 7.3.1. Scenario 1: Households and Service Provider in the Trust Boundary

Table 1 lists the amortized time and disk consumption of the operations of roles in Scenario 1 (Figure 3) that are independent of the number of households. The households transfer data to the service provider and wait for the analyzed results. The service provider is responsible for generating the keys and the work of encryption and decryption. The analysts perform analytical computations over the encrypted data.

**Figure 11:**
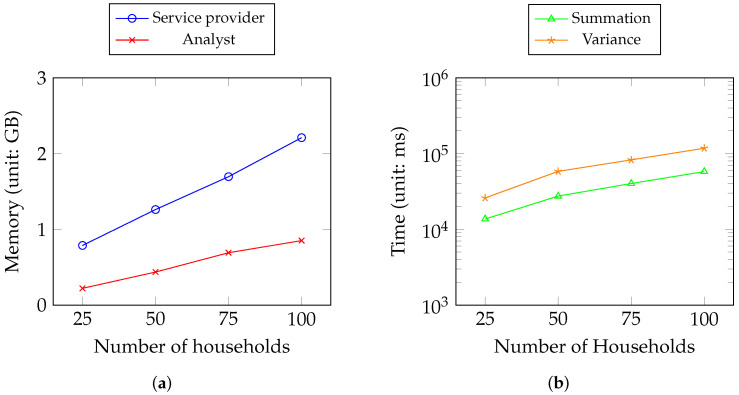
Memory and time consumption in Scenario 1. (**a**) Memory consumption of the service provider and the analyst; (**b**) time consumption of computation metrics of the analyst.

As shown in Figure 11, the memory and time consumption are recorded for Scenario 1. Figure 11a shows the memory consumption of two roles (i.e., the service provider and the analyst) ( Figure 3). Figure 11b shows the time consumption for the summation and variance metrics of the analyst.

Table 1 shows that all operations require less than 100 milliseconds except for rotation key generation. The rotation key generated requires the biggest disk storage depending on the size of the security parameter we use. As the key generation takes place once, this is still acceptable. For third-party analysts, multiplication and rotation account for most of the HE computation, as expected. Figure 11 shows that the memory of the service almost linearly increases because it needs to encrypt and decrypt the households’ data other than the keys’ generation. Therefore, the time consumption for metrics by analysts is acceptable with the increase in households considering the consumption data size per day.

#### 7.3.2. Scenario 2: Households and Service Provider in the Trust Boundary

Table 2 shows the amortized time and disk consumption of the operations of roles in Scenario 2 (Figure 5) that are independent of the number of households. The households encrypt data using the public key, transfer ciphertexts to the main household and wait for the analyzed results. The service provider is the medium for data transmission between households and third-party analysts back and forth. The analysts perform analytical computations over the encrypted data.

**Figure 12:**
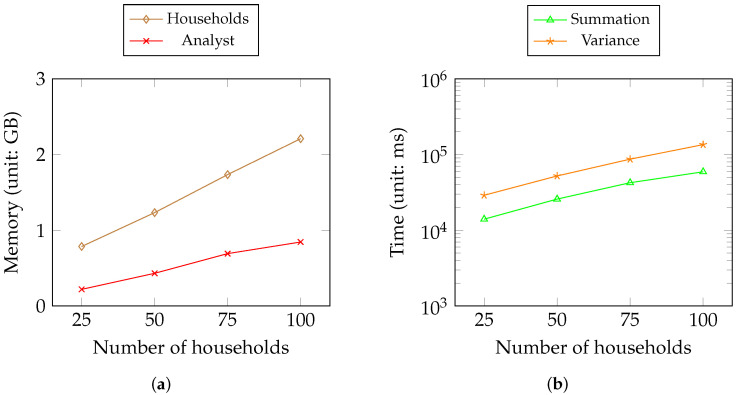
Memory and time consumption in Scenario 2. (**a**) Memory consumption of the households (including the main household) and the analyst; (**b**) time consumption of computation metrics of the analyst.

Figure 12 lists the memory and time consumption for Scenario 2. Figure 12a shows the memory consumption of two roles (i.e., the service provider and the analyst) in the scenario in Figure 5. Figure 12b shows the time consumption for the summation and variance metrics of the analyst.

Table 2 shows that all operations require less than 150 ms except for rotation key generation, which is a little slower than that in Scenario 1 because households usually have fewer memory resources than the service provider. The rotation key generated requires the biggest disk storage depending on the size of the security parameter we use. As the key generation takes place once, this is still acceptable. For third-party analysts, multiplication and rotation account for most of the HE computation, as expected. Figure 12 shows that the memory of the households almost linearly increases because they need to encrypt and decrypt all households’ data and generate the keys. Therefore, the time consumption for metrics by analysts is acceptable with the increase in households considering the consumption data size per day.

#### 7.3.3. Scenario 3: Households and Service Provider in the Trust Boundary

Table 3 lists the amortized time and disk consumption of the operations of roles in Scenario 3 (Figure 7) that are independent of the number of households. In detail, multiparty homomorphic encryption includes a multiparty computation protocol by collectively generating HE keys. Except for the secret key entirely produced by each household, other keys’ generations are split into two parts: the time of computing its share locally for each household (i.e., labeled with “local”) and the time of combination of these shares by the service provider (i.e., labeled with “cloud”). After keys are generated, they will be distributed appropriately. Public keys are assigned to households, and evaluation keys are sent to third-party analysts. The households encrypt data using the public key, transfer ciphertexts to the main household, and wait for the analyzed results. The service provider is the medium for data transmission between households and third-party analysts back and forth. The analysts perform analytical computations over the encrypted data.

**Figure 13:**
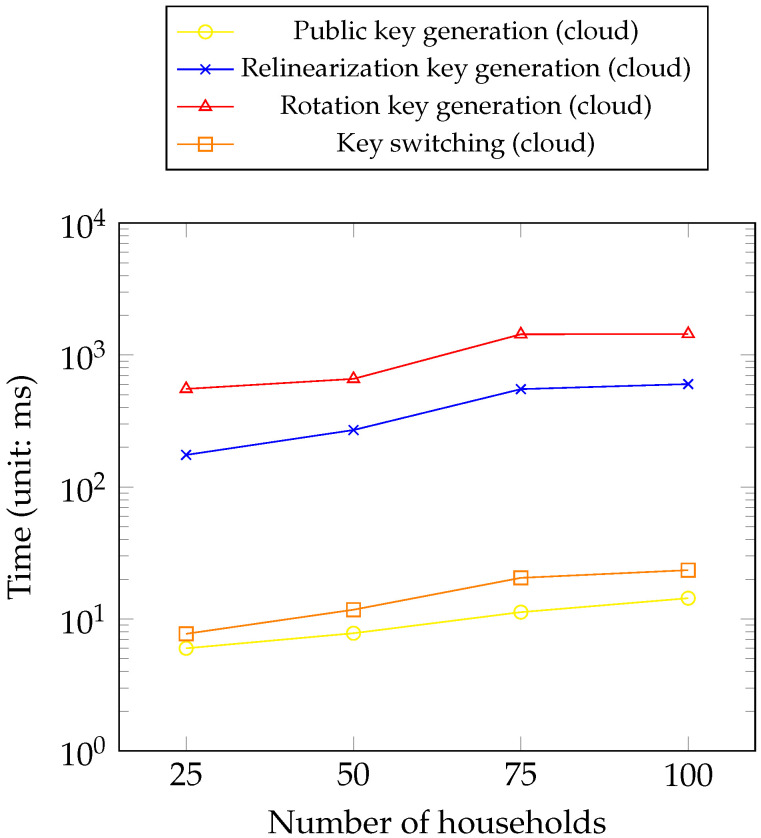
Amortized time consumption of the combination part (i.e., the service provider) of an MPC protocol for collective keys’ generation.

Figure 13 shows the time consumption for the cloud parts of the MPC protocol in the service provider concerning different numbers of households (e.g., 25, 50, 75, and 100).

**Figure 14:**
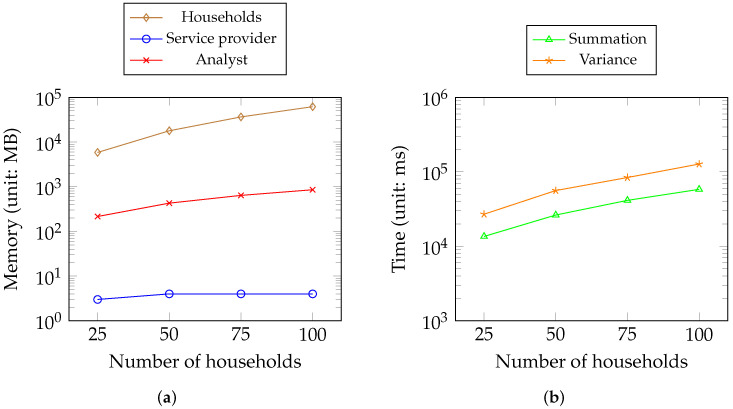
Memory and time consumption in Scenario 3. (**a**) Memory consumption of the households, the service provider, and the analyst; (**b**) time consumption of computation metrics of the analyst.

Figure 14 displays the memory and time consumption for Scenario 3. In Figure 14a, we depict the memory consumption of two roles (i.e., the service provider and the analyst) for the scenario in Figure 7. Figure 14b shows the time consumption for the summation and variance metrics of the analyst.

Table 3 shows that all operations locally require less than 250 ms except for rotation key generation. The generated rotation key requires the most disk storage depending on the size of the security parameter we use. As the key generation takes place once, this is still acceptable. For third-party analysts, multiplication and rotation account for most of the HE computation, as expected. Figure 13 shows the time consumption of the cloud part of the MPC protocol on the service provider’s side. It shows that the time required increases enormously with increasing number of households. The rotation key again accounts for most of the time, which has the same pattern among all the keys. Figure 14 shows that the memory of the households and the service provider notably increases while the memory of the analysts remains stable. The households account for most of the memory consumption because they are responsible for key generation and encryption. Therefore, the time consumption for metrics by analysts is acceptable with the increase in households considering the consumption data size per day.

### 7.4. Performance Comparison with Related Work

The HE library we used, Lattigo [17], provides a competitive performance compared with other common HE libraries, such as Microsoft SEAL [18], which is discussed in [59]. The performance of HE schemes is not constant and varies based on factors such as security parameters, plaintext and ciphertext sizes, and the underlying hardware and software implementations. Even for the same CKKS scheme, implementations of different libraries also matter. Therefore, it is difficult to provide exact cost comparisons for primitive operations such as encryption, decryption, addition, and multiplication in general. However, we can still provide a meaningful comparison between the results we measured in our proposed framework and the results from [60] (8-core CPU and 16 GB RAM), as shown in Table 4. We utilized the results from Scenario 1, where the encryption and the decryption operations were measured with a Windows laptop (1.90 GHz CPU and 16 GB RAM) for Scenario 1. The Lattigo* column provides from the results of this study.

As mention in Section 7.2, we chose our security parameter set: $Log\left(N\right)=14,Log\left(Q\right)=438,Log\left(Slots\right)=14,DefaultScale=2^{34}$, which is equivalent to the parameter set of $N=16,384,\log_{2}q$ for CKKS in [60]. The comparison shows that the amortized time of primitive HE operations (i.e., encryption and decryption) are equal in the order of magnitude for Lattigo [17] and SEAL [18].

## 8. Discussion

### 8.1. Type 1 Adversaries

In all three scenarios described above, type 1 adversaries at the analysts’ point fall outside the trust boundary (TB). In this way, they may perform malicious activities, such as deliberately performing erroneous computations. We can address this issue in several ways. First, we can accept this risk and assume that the analysts are honest but curious. Under this assumption, the analysts are interested in the data of the customers but do not perform any malicious activities such as deliberately making erroneous computations because they have a reputation to uphold; if they perform malicious activities, we reason that these activities will be eventually identified, and the service providers will no longer work with these analysts.

The service provider can also address this issue by outsourcing the same data to multiple third-party analysts for computation. Then, we assume that the result produced by most analysts is correct. This way, we can reduce the threat of type 1 adversaries and the possibility of an accidentally incorrect computation by one of the analysts. However, one drawback of this approach is the increased costs and time to calculate the result due to using multiple analysts for each customer.

### 8.2. Type 2 Adversaries

In Scenarios 2 and 3, type 2 adversaries at the service provider’s point fall outside the trust boundary (TB). Although we apply the same assumptions that they are honest but curious, we must consider the possibility of them behaving maliciously. Some possible malicious activities include tampering with the data by performing computations to inflate the customers’ actual usage, so they are billed for more resources than they consumed. Furthermore, a type 2 adversary might collaborate with a type 1 adversary. Therefore, if we want a complete data integrity guarantee without making assumptions about the honest but curious nature of type 1 and 2 adversaries, we must integrate a separate data integrity-preserving technology into our framework.

### 8.3. Integrity Preservation

It may be possible to use distributed ledger technology (DLT) to preserve the integrity of the customer’s data throughout the SMS data flow, providing a solution to the concerns above. In a blockchain-based DLT system, each modification to a piece of data is recorded as an immutable transaction, ensuring that the data cannot be tampered with without breaking the chain and being noticed. However, while the blockchain guarantees data integrity, it is not completely impervious to attacks or vulnerabilities [61]. Therefore, further research is required to establish a method to integrate the DLT with our framework.

### 8.4. Computation Metrics

The metrics we use for HE computation are simple and easy to implement, while third-party analysts may perform complex computations that require more advanced metrics for analysis, such as load forecasting, which can be very challenging. As of now, code implementations of the current HE library (e.g., Lattigo [17] and SEAL [18]) use primitive operations, such as addition, subtraction, multiplication, and rotation. There are significant limitations for more advanced computation metrics. Hence, not all metrics can be converted into HE computations. However, some parts of the metrics’ calculation can be transferred into HE computations and be used for the whole metrics to enhance privacy. For instance, if a complex metric includes summation and variance, then the results of the summation and variance by HE can be returned to analysts for the rest of the calculation of that complex metric.

### 8.5. Security Level of the Proposed Framework

The security level of HE schemes refers to the protection they can provide against adversaries’ attacks. The choice of the security level of 128 bits, 192 bits, or 256 bits is the length of the HE encryption keys. Thus, a longer key length requires more keys an attacker needs to try to break the encryption. However, a higher security level may increase the computational overhead, including time consumption, memory consumption, and disk storage of HE keys. So, 128 bits is a good choice for balancing HE security and HE performance for most applications, which is why it is a consensus among the HE community, as listed in [52]. As for its quantum resistance [48], HE is based on the hardness of the RLWE problem, which has no evidence of being able to be easily attacked by a quantum computer for now.

### 8.6. Identity-Based Encryption

In the proposed framework, we utilize a common PKI depending on CA and TLS protocol to keep the communication channel between each entity safe. However, it relies on distributing the public key of the trusted entities in the SMS. Alternatively, we can introduce identity-based encryption (IBE) to simplify the key distribution for secure communication, so that the plaintext result can be securely transferred from the service provider to the households.

To use IBE, we can set up a central trusted authority, the private key generator (PKG), to generate users’ private keys. The PKG publishes a master public key and retains a master secret. A user’s public key can be computed from the master public key with its identity. Then, the master secret and a user’s public identity are used together to generate the corresponding private key for that user. Other entities can encrypt messages using the recipient’s public identity, and only the recipient with the corresponding private key can decrypt the messages.

### 8.7. Energy Consumption

We proposed, designed, deployed, and evaluated the privacy-preserving HE framework for SMSs regarding the trade-off between privacy protection and time/disk consumption. However, other aspects in the deployment of HE solutions need to be considered.

One common aspect of HE deployment is energy consumption. In this field, some studies used hardware to accomplish this task. For example, Reis et al. [62] proposed a computing-in-memory-based HE implementation to gain energy savings of between 266.4 times and 532.8 times for homomorphic multiplications (the most expensive HE operation) compared with a CPU-based HE solution. In addition, Lei et al. [63] proposed an energy-efficient accelerator for fully homomorphic encryption that improves the throughput per Watt by 6.3 times compared with that of previous accelerators.

### 8.8. The Complexity of Privacy, Security, and Safety in SMS

Obtaining adequate billing information is quite an ordinary but useful and necessary service in SMSs. Therefore, three aspects of SMS solutions need to be considered: privacy, security, and safety. Privacy and security are well protected based on our proposed framework. We also have some assumptions of adversaries listed in the threat model and discussed in Section 8.1 and Section 8.2. The safety [14] of SMSs is a broader perspective and can rely on protecting the physical devices, adhering to electromagnetic compatibility standards, and maintaining the reliability of the SMS to prevent accidental or malicious manipulation, which could cause disruptions or damage to the system.

## 9. Conclusions

This paper proposed a privacy-preserving framework for SMS to protect data privacy by applying HE. The framework utilizes different trust boundaries to analyze HE configurations for various scenarios in practical applications, including data flow, privacy risks, and HE key distribution under each scenario. Furthermore, we adopted simple computation metrics (i.e., summation and variance) for third-party analysts applied in HE schemes and evaluates the feasibility of the proposed framework based on real-world time series data of the smart water metering system. We tested the overheads of the proposed framework. Our results show that the computational overhead is still acceptable from a cost–benefit perspective while ensuring customer data privacy.

## Figures and Tables

**Table 1:** Amortized time and disk consumption of operations independent of the number of households concerning roles in Scenario 1 (operations with an asterisk (*) execute multiple times).

Role	Operation Type	Time (ms)	Disk (KB)
service provider	secret key generation	4.859	6741
	public key generation	14.148	14,249
	relinearization key generation	71.221	90,442
	rotation key generation	1267	1,627,943
	HE decryption *	0.919	-
	HE encryption *	20.620	-
household	-	-	-
analyst	HE addition/subtraction *	0.732	-
	HE multiplication *	29.228	-
	HE rotation *	31.596	-

**Table 2:** Amortized time and disk consumption of operations independent of the number of households concerning roles in Scenario 2 (operations with an asterisk (*) execute multiple times).

Role	Operation Type	Time (ms)	Disk (KB)
household	secret key generation	10.616	6901
	public key generation	31.389	14,589
	relinearization key generation	143.726	92,612
	rotation key generation	2508	1,667,020
	HE decryption *	1.948	-
	HE encryption *	46.601	-
service provider	-	-	-
analyst	HE addition/subtraction *	0.732	-
	HE multiplication *	31.181	-
	HE rotation *	30.743	-

**Table 3:** Amortized time and disk consumption of operations independent of the number of households concerning roles in Scenario 3 (operations with an asterisk (*) execute multiple times).

Role	Operation Type	Time (ms)	Disk (KB)
household	secret key generation	12.912	6772
	public key generation (local)	9.795	14,264
	relinearization key generation (local)	208.277	90,174
	rotation key generation (local)	649.591	1,645,357
	key switching (local) *	43.361	-
	HE encryption *	21.958	-
service provider	HE decryption *	0.814	-
analyst	HE addition/subtraction *	0.776	-
	HE multiplication *	30.423	-
	HE rotation *	32.515	-

**Table 4:** Amortized time for encryption and decryption in CKKS scheme.

Operation Type	HE Parameters		HE Library			
	*n*	$\log_{2}q$	Palisade	HELib	SEAL	Lattigo*
Encryption	16,384	438	23.183	12.581	19.344	20.620
Decryption	16,384	438	13.776	183.254	1.166	0.919

## Data Availability

Not applicable.

## Abbreviations

The following abbreviations are used in this manuscript:

SMS	Smart Metering System
SWM	Smart Water Metering
LWE	Learning with Errors
RLWE	Ring Learning with Errors
HE	Homomorphic Encryption
FHE	Fully Homomorphic Encryption
MHE	Multi-Party Homomorphic Encryption
MPC	Multi-Party Computation
CPK	Collective Public Key
CEK	Collective Evaluation Key
TB	Trust Boundary
CA	Certificate Authority
PKI	Public Key Infrastructure
DoS	Denial of Service
MitM	Man-in-the-Middle
TLS	Transport Layer Security
DP	Differential Privacy
LDP	Local Differential Privacy
TEE	Trusted Execution Environment
AMI	Advanced Metering Infrastructure
DLT	Distributed Ledger Technology
KB	Kilobyte
IND-CPA	Indistinguishability under the Chosen Plaintext Attack
IBE	Identity-based Encryption
PKG	Private Key Generator

## References

[1] Mbungu N.T., Bansal R.C., Naidoo R.M., Bettayeb M., Siti M.W., Bipath M. (2020). A dynamic energy management system using smart metering. Appl. Energy.

[2] Elsisi M., Mahmoud K., Lehtonen M., Darwish M.M. (2021). Reliable industry 4.0 based on machine learning and IOT for analyzing, monitoring, and securing smart meters. Sensors.

[3] Luciani C., Casellato F., Alvisi S., Franchini M. (2019). Green smart technology for water (GST4Water): Water loss identification at user level by using smart metering systems. Water.

[4] Loureiro D., Vieira P., Makropoulos C., Kossieris P., Ribeiro R., Barateiro J., Katsiri E. (2014). Smart metering use cases to increase water and energy efficiency in water supply systems. Water Sci. Technol. Water Supply.

[5] Chakraborty S., Das S., Sidhu T., Siva A. (2021). Smart meters for enhancing protection and monitoring functions in emerging distribution systems. Int. J. Electr. Power Energy Syst..

[6] Gellings C.W. (2020). The Smart Grid: Enabling Energy Efficiency and Demand Response.

[7] Hledik R. (2009). How green is the smart grid?. Electr. J..

[8] Asghar M.R., Dán G., Miorandi D., Chlamtac I. (2017). Smart meter data privacy: A survey. IEEE Commun. Surv. Tutor..

[9] Lu N., Du P., Guo X., Greitzer F.L. Smart meter data analysis. Proceedings of the PES T&D 2012.

[10] Jawurek M., Johns M., Kerschbaum F. Plug-in privacy for smart metering billing. Proceedings of the International Symposium on Privacy Enhancing Technologies Symposium.

[11] Xue K., Yang Q., Li S., Wei D.S., Peng M., Memon I., Hong P. (2018). PPSO: A privacy-preserving service outsourcing scheme for real-time pricing demand response in smart grid. IEEE Internet Things J..

[12] Cominola A., Giuliani M., Castelletti A., Rosenberg D.E., Abdallah A.M. (2018). Implications of data sampling resolution on water use simulation, end-use disaggregation, and demand management. Environ. Model. Softw..

[13] Cardell-Oliver R., Wang J., Gigney H. (2016). Smart meter analytics to pinpoint opportunities for reducing household water use. J. Water Resour. Plan. Manag..

[14] Fan Z., Kulkarni P., Gormus S., Efthymiou C., Kalogridis G., Sooriyabandara M., Zhu Z., Lambotharan S., Chin W.H. (2012). Smart grid communications: Overview of research challenges, solutions, and standardization activities. IEEE Commun. Surv. Tutor..

[15] Cardell-Oliver R., Carter-Turner H. Activity-aware privacy protection for smart water meters. Proceedings of the 8th ACM International Conference on Systems for Energy-Efficient Buildings, Cities, and Transportation.

[16] Taïk A., Cherkaoui S. Electrical load forecasting using edge computing and federated learning. Proceedings of the IEEE International Conference on Communications (ICC 2020).

[17] (2022). EPFL-LDS—Tune Insight SA. Lattigo v4. https://github.com/tuneinsight/lattigo.

[18] Microsoft Research (2023). Microsoft SEAL (Release 4.1). https://github.com/Microsoft/SEAL.

[19] Cheon J.H., Kim A., Kim M., Song Y. Homomorphic Encryption for Arithmetic of Approximate Numbers. Proceedings of the 23rd International Conference on the Theory and Applications of Cryptology and Information Security.

[20] Ur-Rehman O., Zivic N., Ruland C. Security issues in smart metering systems. Proceedings of the 2015 IEEE International Conference on Smart Energy Grid Engineering (SEGE).

[21] Albrecht J.P. (2016). How the GDPR will change the world. Eur. Data Prot. L. Rev..

[22] Jin M., Jia R., Kang Z., Konstantakopoulos I.C., Spanos C.J. Presencesense: Zero-training algorithm for individual presence detection based on power monitoring. Proceedings of the 1st ACM Conference on Embedded Systems for Energy-Efficient Buildings.

[23] Priyadarshini I., Sharma R., Bhatt D., Al-Numay M. (2022). Human activity recognition in cyber-physical systems using optimized machine learning techniques. Clust. Comput..

[24] Salomons E., Sela L., Housh M. (2020). Hedging for privacy in smart water meters. Water Resour. Res..

[25] Li Z., Oechtering T.J., Skoglund M. Privacy-preserving energy flow control in smart grids. Proceedings of the 2016 IEEE International Conference on Acoustics, Speech and Signal Processing (ICASSP).

[26] Li N., Qardaji W., Su D. On sampling, anonymization, and differential privacy or, k-anonymization meets differential privacy. Proceedings of the 7th ACM Symposium on Information, Computer and Communications Security.

[27] Alsaid M., Slay T., Bulusu N., Bass R.B. K-anonymity applied to the energy grid of things distributed energy resource management system. Proceedings of the 20th Annual International Conference on Mobile Systems, Applications and Services.

[28] Stegelmann M., Kesdogan D. Gridpriv: A smart metering architecture offering k-anonymity. Proceedings of the 2012 IEEE 11th International Conference on Trust, Security and Privacy in Computing and Communications.

[29] Karopoulos G., Xenakis C., Tennina S., Evangelopoulos S. Towards trusted metering in the smart grid. Proceedings of the 2017 IEEE 22nd International Workshop on Computer Aided Modeling and Design of Communication Links and Networks (CAMAD).

[30] Valadares D.C.G., Sobrinho Á.A.D.C.C., Perkusich A., Gorgonio K.C. (2021). Formal verification of a trusted execution environment-based architecture for IoT applications. IEEE Internet Things J..

[31] Kirschbaum M., Plos T., Schmidt J.M. On secure multi-party computation in bandwidth-limited smart-meter systems. Proceedings of the 2013 International Conference on Availability, Reliability and Security.

[32] Danezis G., Fournet C., Kohlweiss M., Zanella-Béguelin S. Smart meter aggregation via secret-sharing. Proceedings of the first ACM Workshop on Smart Energy grid Security.

[33] Gai N., Xue K., Zhu B., Yang J., Liu J., He D. (2022). An efficient data aggregation scheme with local differential privacy in smart grid. Digit. Commun. Netw..

[34] Gentry C. Fully homomorphic encryption using ideal lattices. Proceedings of the 41st Annual ACM Symposium on Theory of Computing.

[35] Tonyali S., Saputro N., Akkaya K. Assessing the feasibility of fully homomorphic encryption for smart grid ami networks. Proceedings of the 2015 Seventh International Conference on Ubiquitous and Future Networks.

[36] Abreu Z., Pereira L. (2022). Privacy protection in smart meters using homomorphic encryption: An overview. Wiley Interdiscip. Rev. Data Min. Knowl. Discov..

[37] Zhang X.M., Grolinger K., Capretz M.A., Seewald L. Forecasting residential energy consumption: Single household perspective. Proceedings of the 2018 17th IEEE International Conference on Machine Learning and Applications (ICMLA).

[38] Stavroulakis P., Stamp M. (2010). Handbook of Information and Communication Security.

[39] Alabdulatif A., Kumarage H., Khalil I., Atiquzzaman M., Yi X. (2017). Privacy-preserving cloud-based billing with lightweight homomorphic encryption for sensor-enabled smart grid infrastructure. IET Wirel. Sens. Syst..

[40] Castelluccia C., Chan A.C., Mykletun E., Tsudik G. (2009). Efficient and provably secure aggregation of encrypted data in wireless sensor networks. ACM Trans. Sens. Netw. (TOSN).

[41] Azaza M., Wallin F. (2017). Smart meter data clustering using consumption indicators: Responsibility factor and consumption variability. Energy Procedia.

[42] Xu C., Li W., Yu M., Xu J., Liu J., Wang Y., Zhu L. A correlation sorting-LSTM method for high accuracy short-term load forecasting based on smart meter data. Proceedings of the 2020 7th International Conference on Information, Cybernetics, and Computational Social Systems (ICCSS).

[43] Karapanos N., Capkun S. On the effective prevention of {TLS} man-in-the-middle attacks in web applications. Proceedings of the 23rd USENIX Security Symposium (USENIX Security 14).

[44] Priyadarshini I., Kumar R., Sharma R., Singh P.K., Satapathy S.C. (2021). Identifying cyber insecurities in trustworthy space and energy sector for smart grids. Comput. Electr. Eng..

[45] Sanjab A., Saad W. (2016). Data injection attacks on smart grids with multiple adversaries: A game-theoretic perspective. IEEE Trans. Smart Grid.

[46] Mouchet C., Troncoso-Pastoriza J., Bossuat J.P., Hubaux J.P. (2021). Multiparty homomorphic encryption from ring-learning-with-errors. Proc. Priv. Enhancing Technol..

[47] Lyubashevsky V., Peikert C., Regev O. (2013). On ideal lattices and learning with errors over rings. J. ACM (JACM).

[48] Peikert C. How (not) to instantiate ring-LWE. Proceedings of the 10th International Conference of the Security and Cryptography for Networks (SCN 2016).

[49] Cheon J.H., Hong S., Kim D. (2020). Remark on the Security of Ckks Scheme in Practice. https://eprint.iacr.org/2020/1581.

[50] Li B., Micciancio D. On the security of homomorphic encryption on approximate numbers. Proceedings of the 40th Annual International Conference on the Theory and Applications of Cryptographic Techniques Advances in Cryptology (EUROCRYPT 2021).

[51] Maringer G., Fritzmann T., Sepúlveda J. The influence of LWE/RLWE parameters on the stochastic dependence of decryption failures. Proceedings of the 22nd International Conference of the Information and Communications Security (ICICS 2020).

[52] Chase M., Chen H., Ding J., Goldwasser S., Gorbunov S., Hoffstein J., Lauter K., Lokam S., Moody D., Morrison T. (2017). Security of Homomorphic Encryption. chrome-extension://efaidnbmnnnibpcajpcglclefindmkaj/https://www.microsoft.com/en-us/research/wp-content/uploads/2018/01/security_homomorphic_encryption_white_paper.pdf.

[53] Hellman M. (1976). New directions in cryptography. IEEE Trans. Inf. Theory.

[54] Miller V.S. (1986). Use of Elliptic Curves in Cryptography.

[55] Garman J. (2003). Kerberos: The Definitive Guide: The Definitive Guide.

[56] Karthikeyan G., Heiss S. Pki and user access rights management for opc ua based applications. Proceedings of the 2018 IEEE 23rd International Conference on Emerging Technologies and Factory Automation (ETFA).

[57] Rescorla E. (2018). The Transport Layer Security (TLS) Protocol Version 1.3; RFC 8446. https://www.rfc-editor.org/info/rfc8446.

[58] (2020). DAIAD. Smart Water Meter Consumption Time Series. https://data.hellenicdataservice.gr/dataset/78776f38-a58b-4a2a-a8f9-85b964fe5c95.

[59] Mouchet C.V., Bossuat J.P., Troncoso-Pastoriza J.R., Hubaux J.P. Lattigo: A multiparty homomorphic encryption library in go. Proceedings of the 8th Workshop on Encrypted Computing and Applied Homomorphic Cryptography.

[60] Marković M., Vuletic P.V. (2021). Performance Comparison of Homomorphic Encryption Scheme Implementations. chrome-extension://efaidnbmnnnibpcajpcglclefindmkaj/https://www.etran.rs/2021/zbornik/Papers/104_RTI_2.5.pdf.

[61] Yoshihama S., Saito S. Study on Integrity and Privacy Requirements of Distributed Ledger Technologies. Proceedings of the 2018 IEEE International Conference on Internet of Things (iThings) and IEEE Green Computing and Communications (GreenCom) and IEEE Cyber, Physical and Social Computing (CPSCom) and IEEE Smart Data (SmartData).

[62] Reis D., Takeshita J., Jung T., Niemier M., Hu X.S. (2020). Computing-in-memory for performance and energy-efficient homomorphic encryption. IEEE Trans. Very Large Scale Integr. (VLSI) Syst..

[63] Jiang L., Lou Q., Joshi N. Matcha: A fast and energy-efficient accelerator for fully homomorphic encryption over the torus. Proceedings of the 59th ACM/IEEE Design Automation Conference.

